# Understanding the Social Mechanism of Cancer Misinformation Spread on YouTube and Lessons Learned: Infodemiological Study

**DOI:** 10.2196/39571

**Published:** 2022-11-14

**Authors:** Ho Young Yoon, Kyung Han You, Jung Hye Kwon, Jung Sun Kim, Sun Young Rha, Yoon Jung Chang, Sang-Cheol Lee

**Affiliations:** 1 Division of Communication and Media Ewha Womans University Seoul Republic of Korea; 2 Department of Media and Communication Studies Jeonbuk National University Jeonju Republic of Korea; 3 Division of Hemato-Oncology Department of Internal Medicine Chungnam National University Sejong Hospital Sejong-Si Republic of Korea; 4 Division of Hematology and Oncology Department of Internal Medicine, College of Medicine Chungnam National University Daejeon Republic of Korea; 5 Daejeon Regional Cancer Center Daejeon Republic of Korea; 6 Division of Medical Oncology Yonsei University College of Medicine Seoul Republic of Korea; 7 Yonsei Cancer Center Yonsei University Health System Seoul Republic of Korea; 8 Division of Cancer Control and Policy, National Cancer Center Korea Department of Cancer Control and Population Health National Cancer Center Graduate School of Cancer Science and Policy Goyang Republic of Korea; 9 Division of Hematology and Oncology Department of Internal Medicine Soonchunhyang University Hospital Cheonan Cheonan Republic of Korea

**Keywords:** cancer misinformation, social media health misinformation, fenbendazole, self-administration, complex contagion, YouTube, social media factual information delivery strategy

## Abstract

**Background:**

A knowledge gap exists between the list of required actions and the action plan for countering cancer misinformation on social media. Little attention has been paid to a social media strategy for disseminating factual information while also disrupting misinformation on social media networks.

**Objective:**

The aim of this study was to, first, identify the spread structure of cancer misinformation on YouTube. We asked the question, “How do YouTube videos play an important role in spreading information about the self-administration of anthelmintics for dogs as a cancer medicine for humans?” Second, the study aimed to suggest an action strategy for disrupting misinformation diffusion on YouTube by exploiting the network logic of YouTube information flow and the recommendation system. We asked the question, “What would be a feasible and effective strategy to block cancer misinformation diffusion on YouTube?”

**Methods:**

The study used the YouTube case of the self-administration of anthelmintics for dogs as an alternative cancer medicine in South Korea. We gathered Korean YouTube videos about the self-administration of fenbendazole. Using the YouTube application programming interface for the query “fenbendazole,” 702 videos from 227 channels were compiled. Then, videos with at least 50,000 views, uploaded between September 2019 and September 2020, were selected from the collection, resulting in 90 videos. Finally, 10 recommended videos for each of the 90 videos were compiled, totaling 573 videos. Social network visualization for the recommended videos was used to identify three intervention strategies for disrupting the YouTube misinformation network.

**Results:**

The study found evidence of complex contagion by human and machine recommendation systems. By exposing stakeholders to multiple information sources on fenbendazole self-administration and by linking them through a recommendation algorithm, YouTube has become the perfect infrastructure for reinforcing the belief that fenbendazole can cure cancer, despite government warnings about the risks and dangers of self-administration.

**Conclusions:**

Health authorities should upload pertinent information through *multiple* channels and should exploit the existing YouTube recommendation algorithm to disrupt the misinformation network. Considering the viewing habits of patients and caregivers, the direct use of YouTube hospital channels is more effective than the indirect use of YouTube news media channels or government channels that report public announcements and statements. Reinforcing through multiple channels is the key.

## Introduction

### Cancer Misinformation and Social Media

Cancer misinformation on social media has piqued the interest of health care professionals. Misinformation is defined as information that causes people to “hold inaccurate factual beliefs” [1]. Studies found that nearly one-third of cancer content on social media was misinformation [2,3] and that 76.9% of it contained harmful information [3]. Cancer misinformation can significantly reduce a patient’s chances of survival. It may lead to a delay in receiving appropriate treatment, the pursuit of unproven alternative therapies, or the rejection of the currently prescribed treatment [2]. Beyond the disintegration of trust between patients, carers, and physicians, the detrimental impacts of disinformation extend to psychological and mental health [2-5].

Indeed, misinformation is one of the most serious negative consequences of social media. False news spreads much “further, faster, deeper, and more broadly” than the truth on social media [6]. The novelty of misinformation grabs the immediate attention of humans, resulting in human-mediated message delivery. The echo chamber induced by the recommendation algorithm amplifies the spread of inaccurate information among individuals with similar interests [7]. Given that caregivers and patients’ family members frequently associate the information community with social support and develop a strong interest in new treatment information [8-10], social media provides the optimal environment for information dissemination, combining human and nonhuman algorithm-driven exposure and connections. This is of particular concern for physicians, as several studies have shown that people are more receptive to misinformation than accurate information [2,4,10].

Thus, it is imperative that public health agencies and health professionals advocate for proactive measures to offset the detrimental consequences of social media misinformation [10-13]. Numerous solutions have been proposed, including digital literacy education [4], accurate information provision [11,14], and media campaigning [15]. While these solutions seek to provide “true” information, rarely have studies been done on concrete and effective ways to disrupt the flow of misinformation on social media, particularly based on an analysis of the current social media information flow network. As a result, a knowledge gap exists between policy implementation and the list of required actions [16].

This may be attributed to the communication pattern of cancer misinformation. Misinformation is frequently mixed with true information in everyday health communication [17]. This mode of communication has rendered it incapable of scrutinizing new information, because information is not only the mixture of true and false but of old and new. As a result, studying the spread of cancer misinformation requires a clearly visible case that can track and reconstruct the pattern of communication.

### Complex Contagion of Health Behaviors in Social Media

Adoption of behaviors frequently manifests as a complex contagion: the spread of collective behaviors requiring contact with multiple sources of activation [18]. Complex contagion occurs when social reinforcement influences the transmission of behaviors, beliefs, and attitudes. Unlike simple contagion, in which exposure results in immediate transmission, complex contagion requires social legitimation, credibility, and behavioral complementarity due to uncertainty [18,19]. Studies found that adopting health-related behaviors, such as smoking, exercise, and antivaccine beliefs, are proven to follow the complex contagion mechanism [20-22]. The key point here is not repeated exposure to a single source, but the exposure to multiple sources for social confirmation and reinforcement [18].

Studies have discovered that social media is one of the perfect environments for observing complex contagion [23-25]. Clustered communities in social media serve as peer-to-peer communication and information dissemination networks [25-27]. Social media research frequently reveals patterns of shared exposure to common stimuli [27], but not all patterns are identical. If complex contagion is viewed as the direct link between social media users, it is social cohesion that binds people together in a networked group, often seen in Facebook [28,29]. If the contagion is based on exposure between individuals, regardless of connection between users, then it is contagion via the network structure that enables access to the same source of exposure, typically found in YouTube through its recommendation algorithm [30,31]. The continuous flow of relevant and engaging YouTube videos, as well as the linking of video content via YouTube’s recommendation system [17,32], creates the environment conducive to social reinforcement, a necessary requirement for complex contagion.

### Research Context: Fenbendazole Self-administration on YouTube

On September 3, 2019, a Korean-language YouTube video portraying Joe Tippens, who claimed to be entirely cured of his cancer after self-administration of fenbendazole, was uploaded. This video had a total of 2.4 million view counts and 33,702 likes as of September 21, 2021, and was an instant hit among cancer patients and caregivers in South Korea [33]. On September 20, 2019, news outlets reported that pharmacies were out of stock of fenbendazole tablets [34]. According to the Korean government, fenbendazole sales totaled approximately 229,000 tablets in September 2019, which was 5 times the quantity sold from January to August 2019. In November 2019, around 403,000 tablets were sold [35]. The media also grabbed public attention by reporting about a patient with cancer who was a comedian who publicly proclaimed that he would disclose his status following self-administration of fenbendazole. The Ministry of Food and Drug Safety of South Korea issued a caution on the use of fenbendazole for cancer treatment on October 28, 2019 [36]. The National Cancer Center Korea indicated in January 2020 that no clinical trials with fenbendazole were planned, adding that “it is not worthwhile to pursue” [37]. However, YouTube videos describing personal experiences with self-administration of fenbendazole were continuously released across patient and caregiver communities

### Research Questions

Given the research context, this study attempted to address the following research questions: (1) What are the characteristics of the YouTube cancer misinformation network regarding the self-administration of fenbendazole? More specifically, (2) is there evidence to support the complex contagion process in social media? (3) Does the networking pattern of the YouTube misinformation network for fenbendazole provide us with useful insights as to why the conventional campaign of disrupting misinformation through news media is less effective? (4) Is there a structural separation of the network between news media providers and user-generated content?

## Methods

### Study Design

We have undertaken unstructured data analysis by collecting data from YouTube. Following the collection of YouTube video data, the data were analyzed using timeline trend analysis, content analysis, and network analysis. These are common strategies applied to unstructured big data analysis. Indeed, mining unstructured online data and analyzing them to discover patterns is a basic technique for big data analytics [38]. The detailed process of data collection, network data processing, and data analysis is depicted in Multimedia Appendix 1.

### YouTube Data Collection

We used Google’s YouTube application programming interface (API) to retrieve a list of search query and recommended videos [39] by using a Python program. The API provides the meta information, such as a video title, channel name, the date and time of upload, and number of views, likes, and comments [40].

**Figure 1:**
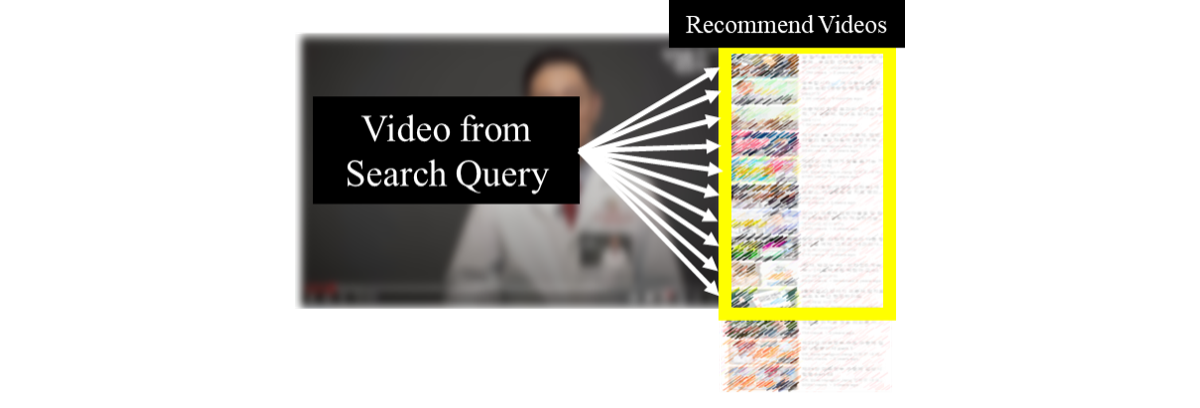
YouTube recommended video interface. Recommended videos are highlighted on the right side. For privacy protection, the screenshot is blurred.

Our data collection strategy was as follows. First, we used the term “fenbendazole” in Korean to download the list of the videos from the YouTube API. Second, videos were included in the analysis if the video views were more than 50,000, uploaded between September 2019 and September 2020. The number 50,000 has been frequently used in the literature to filter relevant and popular YouTube videos [41,42]. Third, we compiled a list of the top 10 recommended videos for each video we searched. Figure 1 depicts the recommended video interface. The top 10 is the number that has been used in the literature for examining recommendation effects [43,44]. Note that this list was not the same list as our API search query for fenbendazole. YouTube’s recommendation algorithm analyzes viewers’ data regarding their viewing habits and uses the data to make recommendations. This does not imply that the YouTube API reflects the preferences of the API key holder. The YouTube API returns the data to the developer that matches the query parameter, such as video, channel, and playlist [39]. It is known that the YouTube API offers results based on popularity rather than individual user desires [45].

### Video Content Analysis

To comprehend the content of the collected fenbendazole YouTube videos, we manually examined the videos that met our selection criteria. Two PhD media researchers participated as coders. The two coders have never viewed fenbendazole YouTube videos. Detailed information about the YouTube videos, such as channel name, video title, and view count, was provided as well as the YouTube video URLs.

The coders coded each video’s channel holders and the viewpoint or attitude toward the self-administration of fenbendazole for cancer treatment. Prior to the coders watching the videos, the channel holders were coded as individuals or institutions. The coders selected subcategory codes for each type of holder after viewing a few videos and engaging in discussion with the research team. We coded individuals as patients, health care professionals, caregivers, and others. The coders further coded whether the holder self-administered fenbendazole if the holder was a patient. Regarding institutions, we categorized the holders as news media, hospitals, and others.

We classified the viewpoint or attitude toward the self-administration of fenbendazole into four subcategories:

1. The “positive” subcategory code was used when the account holder mentioned that she had self-administered fenbendazole or had recommended or sometimes guided others on how to take fenbendazole without physician consultation.
2. The “reserved” subcategory code was used when the account holder mentioned that doctor consultation for fenbendazole self-administration was necessary or that fenbendazole may only be used for people with specific health conditions, such as patients with terminal-stage cancer or when no treatment options remained.
3. The “neutral” subcategory code was used when the video only introduced the fenbendazole case.
4. The “negative” subcategory code was used when the viewpoint was the opposite of the positive viewpoint (ie, the account holder was against the use of fenbendazole).

Finally, our coders reviewed the comments on the videos in order to understand how viewers responded to the videos and to validate their coding by determining whether viewers interpreted the videos as they had been coded.

### Influencer and YouTube Video Recommendation Network

A variety of techniques were used to decipher the misinformation network. First, the timeline of uploaded videos was analyzed, and the influencers, in terms of view count and location in the core of the network, were identified through the network analysis index, *k*-core. Second, network diagrams were drawn in order to locate the influencers in the network. We used the edge list format to convert the list of video searches and recommendations into a network matrix of relational data. In this process, duplicate videos were counted as the value of network ties. The NetDraw program (Analytic Technologies) was then used to draw and to analyze the network data [46]. Although the analysis unit of the collected data was a video, we depicted the network diagram at the channel level for intuitive understanding, as we were particularly interested in who formed the core of the network rather than the role of each video. As the recommendation algorithm of YouTube reflects the quality of video measured by user appreciation, personal preference, and diversity [40], it is logical that the channels at the network’s core exert the most influence on information flow. Through *k*-core analysis and multidimensional scaling (MDS), we were able to determine the network core. *k*-core is an index that identifies a highly cohesive region of the entire graph [47]. When the *k*-core property is combined with the MDS property that clusters network nodes with comparable relationships to other nodes, the *k*-core nodes tend to locate at the center of the network (ie, the core of network). As the majority of YouTube videos are viewed as a result of recommendations [44], the core of the recommendation network is the center of the information cascade in the dissemination of misinformation.

Third, we investigated the network by examining the ego networks of institutions’ channels, such as hospitals and news organizations, to see if they were in the core of the network. The ego network or egocentric network refers to “a network based on a particular individual...comprised of all the relations that a focal agent has with others” [47]. We think the connection to news media and hospitals in the network is important, as trust in these institutions is related to the spread of misinformation [9]. In addition, the news media serve as fact-checking institutions, and hospitals provide scientific health information to counter misinformation. Thus, a comparison of both institutions’ ego networks can help us better comprehend and analyze the networking pattern between traditional media and YouTube viewing habits.

### Ethical Considerations

This study used publicly available data, which are exempt from Institutional Review Board (IRB) approval as stated in Article 16 of the Enforcement Rule of Bioethics and Safety Act of South Korea and Article 13 of its Enforcement Decree. The legislation specifies that publicly available data are waived from IRB review, unless they include collection and recording of sensitive personal information regulated by the Personal Information Protection Act of South Korea. This study did not collect any personal information.

## Results

### Searched and Collected Data

The YouTube API search for the term “fenbendazole” in Korean returned 702 videos from 227 channels. In total, 90 videos received over 50,000 views. The videos with more than 50,000 views accounted for 98% of the total view traffic from 702 videos. Regarding recommended videos, 573 videos were collected because of the recommendation overlaps between the recommendations from 90 videos. The total number of overlapping videos was 36.3% (n=327) of the theoretical maximum number of videos (ie, 90 videos × 10 recommendations each = 900) without any overlap between recommended videos. The overlap percentage between the searched results and the suggested videos was 25.6%, as the suggested videos were not restricted to fenbendazole videos but reflected the users’ other viewed subjects.

### Self-administered Fenbendazole Videos and Opinions From Health Professionals

**Figure 2:**
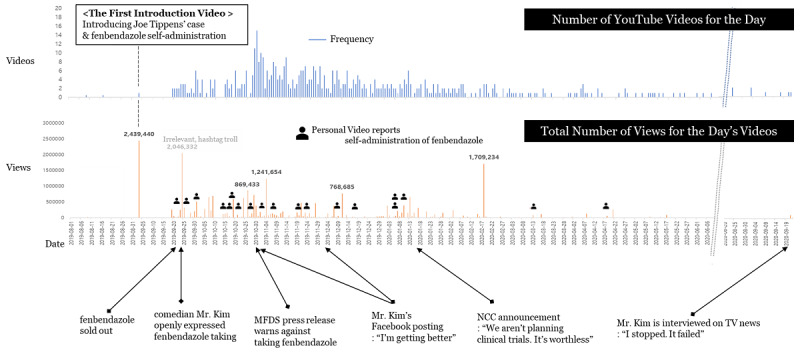
The timeline of fenbendazole YouTube videos. MFDS: Ministry of Food and Drug Safety; NCC: National Cancer Center Korea.

The number of YouTube videos uploaded for the day and the total number of views for the day’s videos are depicted in Figure 2. These videos are the result of an API search query. The graph indicates that a significant number of personal videos talking about self-administration of fenbendazole were uploaded during the first 6 months after Joe Tippens’ case was introduced.

Interestingly, individuals who self-administered fenbendazole continued to appear on YouTube following the news reports about fenbendazole selling out. Although the number of personal videos dealing with the body’s reaction after self-administration was not large, the consistency of video uploads was of crucial importance; it was not repeated exposure to a single source that confirmed people’s perspectives but multiple exposure to diverse sources that confirmed their views. Furthermore, these videos have garnered considerable attention. The top three videos showing responses to self-administered fenbendazole received an average of 215,256 views, which is 4 times the average view count of 50,326 for all 573 videos in the analysis data set. As these videos reported a favorable self-assessment of fenbendazole for pain relief and a fall in tumor markers, the information has virtually become reliable information to viewers. The top 20 most-viewed videos are listed in Table 1; 8 of these are personal testimonials about how fenbendazole improved their symptoms.

In addition, some health professionals on YouTube have not strongly criticized fenbendazole use. This signals to viewers that it would be worth trying because at least it would not be toxic. The comments in these videos referred to these health professionals as “true doctor” [48]. Out of 3 professionals, 2 recommended that patients should consult their doctors before taking fenbendazole (Table 1), but they also described the role of other catalysts, such as vitamins, in aiding in the absorption of the fenbendazole components. As these health professionals are active physicians—one is a physician practicing internal medicine and holds a PhD in chemistry, and the other is the director of the Division of Hematology and Medical Oncology in a general hospital—people interested in the self-administration of fenbendazole interpreted this as a positive signal for taking the medication. For example, the comments by users in the video by internal medicine physicians included the following: “an excellent video to know more details. It helped me making decision between confusing opinions” (ID: ***** Kim, anonymized for privacy protection), “...it was hard to trust physicians but by listening your heartfelt comments, my negative trauma is gone today” (ID: ** tree, anonymized), and “thank you for encouragement as a person taking fenbendazole with colorectal cancer at stage 4” (ID: ***flower, anonymized). One other professional—a clinic owner and radiologist—even advocated for the use of fenbendazole.

**Table 1:** The top 20 YouTube fenbendazole videos.

Date (year-month-day)	Channel	Description^a^	Title summary	Viewpoint^b^	View count^c^, n	Like count^c^, n
2019-09-03	World Village Magazine TV	First video about fenbendazole effect	Introduction of Joe Tippens	Positive	2,439,440	33,702
2020-02-18	VIDEOMUG	News media	News Report of Issue	Neutral	1,708,628	11,829
2019-11-04	Dr. Ezra TV	Health professional	Albendazole Possibility	Reservation	1,151,984	37,337
2019-10-26	JIGUIN	Self-administration	Final-Stage Cancer Symptom	Positive	869,433	9196
2019-12-11	Dr. Ezra TV	Health professional	Parasites That Transmit Cancer	Reservation	660,420	26,729
2020-01-13	Changbal Testing TV	Hashtag troller	Parasite Shock Visual	Positive	600,257	9529
2019-10-29	Dr. Ezra TV	Health professional	Personal Thought	Reservation	521,487	16,738
2019-10-07	JIGUIN	Self-administration	Postfenbendazole Personal Review	Positive	477,383	10,320
2020-01-02	KimCell	Patient taking albendazole (2 tablets)	Metastatic Cancer and Fenbendazole	Positive	387,156	—^d^
2019-10-09	Ahn	Self-administration	Postfenbendazole Personal Review	Positive	382,838	6694
2019-10-30	JIGUIN	Self-administration	Postfenbendazole Personal Review	Positive	382,818	9652
2020-01-10	Sanchae Story	Self-administration	Self-administration of Fenbendazole and Cured	Positive	362,256	6909
2019-10-01	JIGUIN	Self-administration	Postfenbendazole Personal Review	Positive	325,478	5562
2019-11-21	Dr. Ezra TV	Health professional	US Government is Testing Fenbendazole	Partially positive or reservation	299,936	13,376
2019-10-09	MitoTV (doctor)	Health professional	Anticancer Anthelmintics?	Positive	278,706	8120
2019-10-05	JIGUIN	Self-administration	Reply to Comments	Positive	278,395	8838
2020-04-21	Segye Ilbo (newspaper)	News media	News Report on Mr. Kim	Neutral	261,328	1534
2019-10-24	JIGUIN	Self-administration	Cancer Expert View	Positive	258,664	7543
2019-11-28	Changbal Testing TV	Hashtag troller	Parasites and Cancer	Not applicable	248,907	7009
2019-09-23	Dr. Nah’s Medical Talk	Health professional	Expert View on Fenbendazole	Negative or reservation	239,643	3777

^a^The description category is based on the classification of the channel holder.

^b^The viewpoint category is based on the attitude toward the self-administration of fenbendazole.

^c^The count as of September 21, 2021, the date of data collection.

^d^Not reported.

### YouTube Recommendation Network: Creating a Spiral Circle of Positivity for Fenbendazole Self-administration

**Figure 3:**
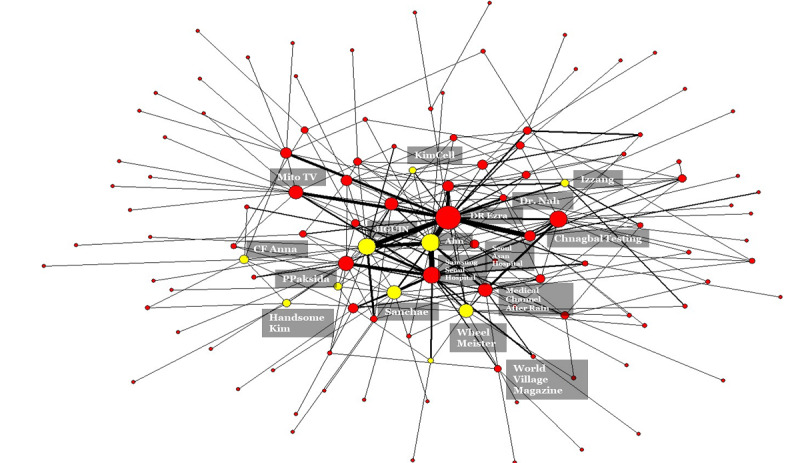
Fenbendazole YouTube video recommendation network between September 2019 and February 2020. The default node color is red. The yellow nodes are the channels where fenbendazole self-administration was conducted. The node size reflects the degree of a node, namely, the number of connections with other nodes. The tie strength reflects the number of repeated recommendation linkages.

Figure 3 displays the recommendation video network during the first 6-month period; the network during the whole year is displayed in Multimedia Appendix 1. The network demonstrates that personal videos after the self-administration of fenbendazole were continuously posted and connected through the recommendation algorithm.

The recommendation network reveals that positive evaluation videos by individual patients and videos by health care professionals synergistically promoted the spread of favorable discourse about fenbendazole. In Figure 3, the first video explaining the positive effect of fenbendazole self-administration on cancer is located outside the network. The central position was taken by one professional video channel—Dr. Ezra TV—which stated that it is preferable for the Korean government to allow people to take fenbendazole in order to collect feasible data rather than warning people, and that it is a sign of capitalism in a capitalist society that a cheap affordable medication would not be invested in for cancer treatment. The Dr. Ezra TV channel was linked to seven video channels about self-administered fenbendazole in a single step, which means that personal videos on the body’s reaction to fenbendazole were directly recommended by the YouTube recommendation system. The channels with the top 20–highest view counts were inextricably linked. For example, the Ahn channel and the JIGUIN channel, along with the Dr. Erza TV channel, were a tightly connected network group, namely, a network clique. These two patient channels reported positive outcomes after the self-administration of fenbendazole in terms of pain reduction and positive blood test results; albeit they did acknowledge that it was unknown whether the effect was primarily due to fenbendazole or was accompanied by other treatments and procedures.

By contrast, the network of recommendations did not include any government or authoritative medical channels. Within the network core, there were two hospital channels; however, it appears that the channels just mirrored users’ YouTube viewing habits. These channels did not include any content on fenbendazole. In fact, these two hospitals are the top two hospitals in terms of the number of cancer patients they treat. News channels played no role in disseminating “true” pertinent information. This becomes obvious when the recommendation network is enlarged to a 1-year time range (Multimedia Appendix 1) in order to visualize the links between the spiral of information and news media, as the initial 6-month recommendation network did not reveal many news media linkages.

**Figure 4:**
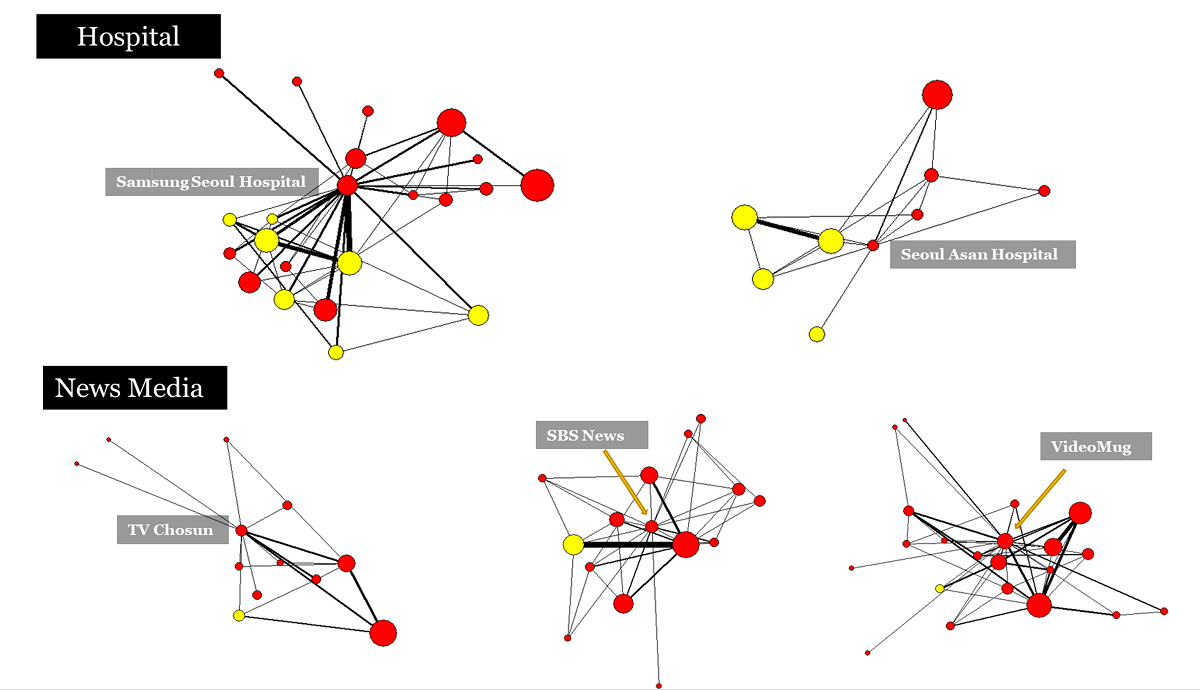
Ego networks of the fenbendazole YouTube recommendation network by institutions between September 2019 and February 2020. The default node color is red. The yellow nodes are the channels where fenbendazole self-administration was conducted. The node size reflects the degree of a node, namely the number of connections with other nodes. The tie strength reflects the number of repeated recommendation linkages.

The ego network of two hospitals and three news media outlets is depicted in Figure 4. The top two diagrams demonstrate how hospital channels were immediately connected to numerous video channels about self-administration of fenbendazole via a single recommendation, whereas news channels were rarely connected to these. In other words, even if accurate information is distributed, patients and caregivers engaged in self-administration are unlikely to be connected to government and other authentic YouTube medical channels.

## Discussion

### Principal Findings

This study delved into one of the cancer misinformation networks on YouTube. By analyzing the data from searched and recommended videos, we found that personal videos about self-administered fenbendazole were continuously uploaded and accumulated over time as if showing promising evidence for the use of fenbendazole as a cancer treatment. In addition, the recommended content network of fenbendazole has become the infrastructure for confirming the audience’s belief and hope in fenbendazole as an alternative cancer medicine. Patients are actively seeking health information over the internet, thereby increasing their self-efficacy in making treatment decisions and altering provider-patient interactions [49-52]. As such, the appearance of supportive professional videos stating that the use of fenbendazole for cancer is scientifically unknown, but possibly helpful, may inspire hope and belief among cancer patients and caregivers who are thinking about self-administering fenbendazole; these videos may also lead patients and caregivers to disregard announcements from the National Cancer Center Korea and the Korean Medical Association.

Unfortunately, the effectiveness of fenbendazole has not been established, and other major adverse effects have been observed [53]. During our investigation, we also found that the YouTube recommendation network was unrelated to credible medical knowledge content. Although some hospital channels were, indeed, connected to the network, it seemed to reflect people’s viewing habits rather than topics related to fenbendazole.

In summary, while the YouTube content and recommendation network served as a substantial information source for complex contagion, medical institutions and government entities were excluded from the network, and no dialogue from them was discovered. This resulted in a breakdown of communication between patients and caregivers, resulting in enormous sales of fenbendazole tablets.

### Strategies to Fight Social Media Cancer Misinformation

Given YouTube’s role as a hub for complex contagion, three strategies to fight against social media cancer misinformation networks are recommended. First, health authorities need to upload a variety of pertinent information through multiple channels. This does not necessarily mean the authorities require multiple channels. They can incorporate existing influencers and other channels. The objective is to have numerous sources of exposure in order to disrupt the cascade of misinformation. A single source will not be sufficient to break the complex contagion dynamic.

Second, it is imperative that health authorities take into account YouTube’s recommendation system, current viewing habits, and information flow network between patients and caregivers. As illustrated in Figure 4, prominent hospital channels may be an option, as stakeholders are already engaged in the channels’ content. Adding a new channel would be ineffective, as it would need to build an audience from scratch.

Third, relying on the news media does not resolve the issue: health authorities must take an active role in resolving social media misinformation. The news media, on the other hand, is frequently constrained by mechanical objectivity and is prone to report both sides of an argument. Furthermore, those who follow the news media are not the target audience for the health authorities’ message. For example, social media is not the first preference of individuals who intend to learn from the news.

### Limitations

While our investigation exposed YouTube’s cancer misinformation network, it is not without limits. To begin, the study’s data were not collected in real time. The real-time data collection process may have resulted in more concrete information flow dynamics between YouTube videos. However, one of the most difficult areas of health communication is the real-time monitoring system for cancer misinformation. It is difficult to classify misinformation in advance, before it spreads on social media, unless a global censorship system is in place to monitor every conversation. The fenbendazole case in this study, for example, demonstrates how the unexpected introduction of a case from a foreign country, such as that of Joe Tippens, prompted reactions among Korean cancer patients and caregivers.

Second, the study is country specific and focused on fenbendazole as a case study. Although we discovered the insight that the spread of cancer misinformation follows complex contagion logic in social media, more evidence is necessary to augment the study’s conclusions. Interviews with people who have taken fenbendazole, in particular, would be helpful in determining the intensity of social media influence.

Third, we concentrated on the videos that had the highest number of views. This excludes individuals who self-administered fenbendazole but received little attention. Fenbendazole was extensively used to the extent that it was sold out at the national level. A future large-scale national-level investigation will further enrich our understanding of social media misinformation.

### Conclusions

This study contributes to the body of knowledge by including practice strategies for combating social media cancer misinformation. By studying the content on YouTube, this study has attempted to close the knowledge gap between what to do and how to do it when it comes to delivery of accurate information via social media. The study proposed a way for campaigning against misinformation and educating people by having health policy authorities use the current network of information flow on YouTube. The study focused on involvement with the actual information flow network rather than implementing a conventional one-way communication strategy, even on social media. This action plan will offer valuable information, especially for people who rely solely on online resources, such as social media, and have limited means of accessing expert health knowledge and information. The study’s recommendation may not be a comprehensive strategy for combating misinformation, but it may be one of the most successful methods for increasing trust between health care practitioners and stakeholders, such as patients and caregivers.

## Appendix

Multimedia Appendix 1 Study design and network diagram.

## Abbreviations

API: application programming interface
IRB: Institutional Review Board
MDS: multidimensional scaling
