# Correction: Understanding the Social Mechanism of Cancer Misinformation Spread on YouTube and Lessons Learned: Infodemiological Study

**DOI:** 10.2196/44334

**Published:** 2022-11-25

**Authors:** Ho Young Yoon, Kyung Han You, Jung Hye Kwon, Jung Sun Kim, Sun Young Rha, Yoon Jung Chang, Sang-Cheol Lee

**Affiliations:** 1 Division of Communication and Media Ewha Womans University Seoul Republic of Korea; 2 Department of Media and Communication Studies Jeonbuk National University Jeonju Republic of Korea; 3 Division of Hemato-Oncology Department of Internal Medicine Chungnam National University Sejong Hospital Sejong-Si Republic of Korea; 4 Division of Hematology and Oncology Department of Internal Medicine, College of Medicine Chungnam National University Daejeon Republic of Korea; 5 Daejeon Regional Cancer Center Daejeon Republic of Korea; 6 Division of Medical Oncology Yonsei University College of Medicine Seoul Republic of Korea; 7 Yonsei Cancer Center, Yonsei University Health System Seoul Republic of Korea; 8 Division of Cancer Control and Policy, National Cancer Center Korea Department of Cancer Control and Population Health National Cancer Center Graduate School of Cancer Science and Policy Goyang Republic of Korea; 9 Division of Hematology and Oncology Department of Internal Medicine Soonchunhyang University Hospital Cheonan Cheonan Republic of Korea

In “Understanding the Social Mechanism of Cancer Misinformation Spread on YouTube and Lessons Learned: Infodemiological Study” (J Med Internet Res 2022;24(11):e39571), the authors made the following changes.

1. The *Acknowledgments* section was inadvertently excluded in the originally published article.

In the corrected version, the following statement has been added under the new *Acknowledgments* section.

This study was supported by the National R&D Program for Cancer Control through the National Cancer Center (NCC) funded by the Ministry of Health & Welfare, Republic of Korea (HA21C0048).

2. In the originally published article, Affiliations 7 and 8 were incorrectly presented as two separate affiliations.

In the corrected version, Affiliation 7 is revised as follows to present the original two affiliations:

Yonsei Cancer Center, Yonsei University Health System, Seoul, Republic of Korea

The updated list of affiliations and its attribution to the authors are as follows:

Ho Young Yoon^1^; Kyung Han You^2^; Jung Hye Kwon^3,4,5^; Jung Sun Kim^3^; Sun Young Rha^6,7^; Yoon Jung Chang^8^; Sang-Cheol Lee^9^

^1^Division of Communication and Media, Ewha Womans University, Seoul, Republic of Korea

^2^Department of Media and Communication Studies, Jeonbuk National University, Jeonju, Republic of Korea

^3^Division of Hemato-Oncology, Department of Internal Medicine, Chungnam National University Sejong Hospital, Sejong-Si, Republic of Korea

^4^Division of Hematology and Oncology, Department of Internal Medicine, College of Medicine, Chungnam National University, Daejeon, Republic of Korea

^5^Daejeon Regional Cancer Center, Daejeon, Republic of Korea

^6^Division of Medical Oncology, Yonsei University College of Medicine, Seoul, Republic of Korea

^7^Yonsei Cancer Center, Yonsei University Health System, Seoul, Republic of Korea

^8^Division of Cancer Control and Policy, National Cancer Center Korea, Department of Cancer Control and Population Health, National Cancer Center Graduate School of Cancer Science and Policy, Goyang, Republic of Korea

^9^Division of Hematology and Oncology, Department of Internal Medicine, Soonchunhyang University Hospital Cheonan, Cheonan, Republic of Korea

The correction will appear in the online version of the paper on the JMIR Publications website on November 25, 2022, together with the publication of this correction notice. Because this was made after submission to PubMed, PubMed Central, and other full-text repositories, the corrected article has also been resubmitted to those repositories.

