# Supplementary figures and images for: Understanding the Social Mechanism of Cancer Misinformation Spread on YouTube and Lessons Learned: Infodemiological Study
Source: J Med Internet Res. 2022 Nov 14;24(11):e39571. doi: 10.2196/39571 (PMC9699593; doi:10.2196/39571)

# Study Design

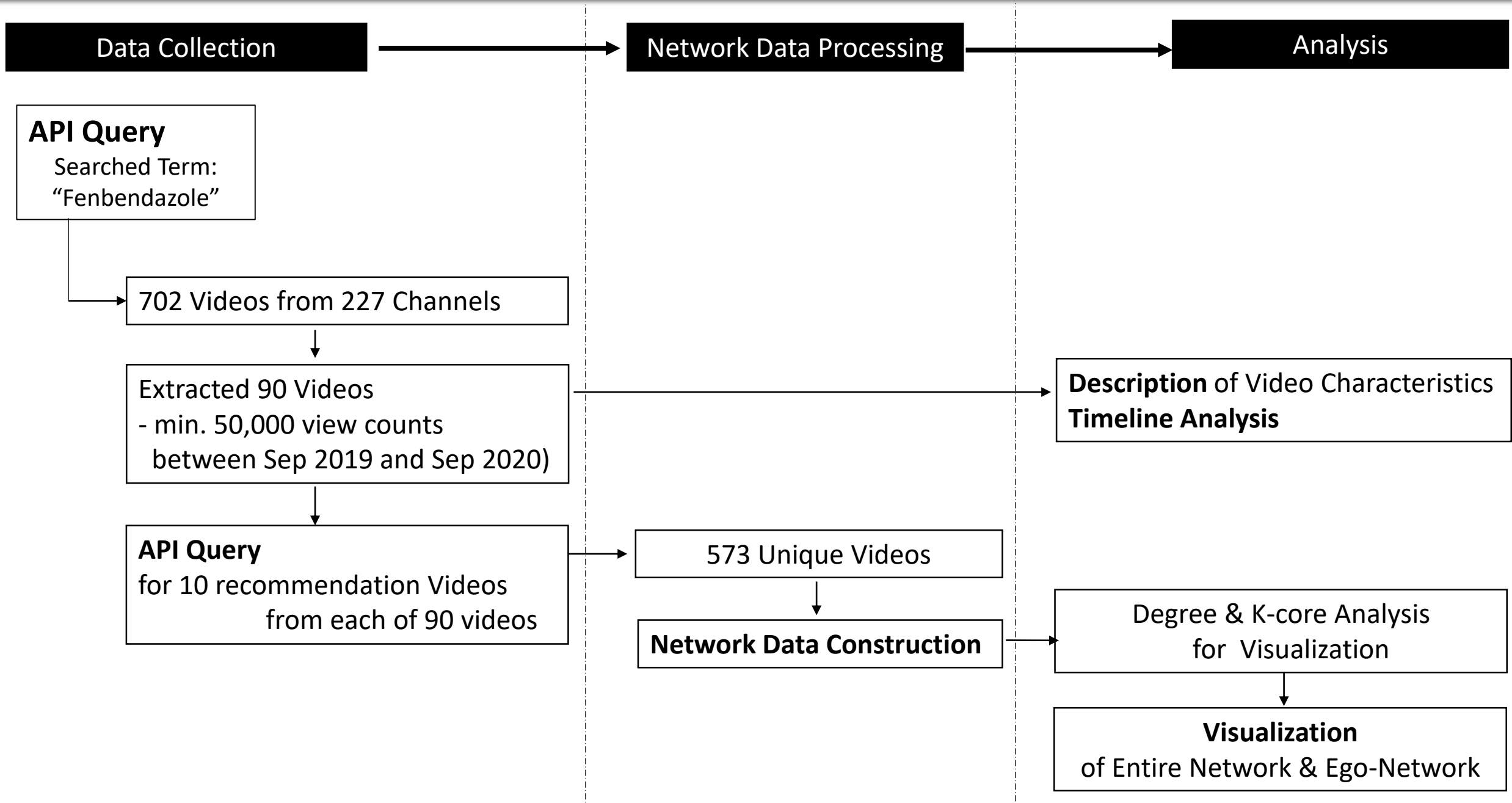

# Network Diagram (Sep 2019 – Sep 2020)

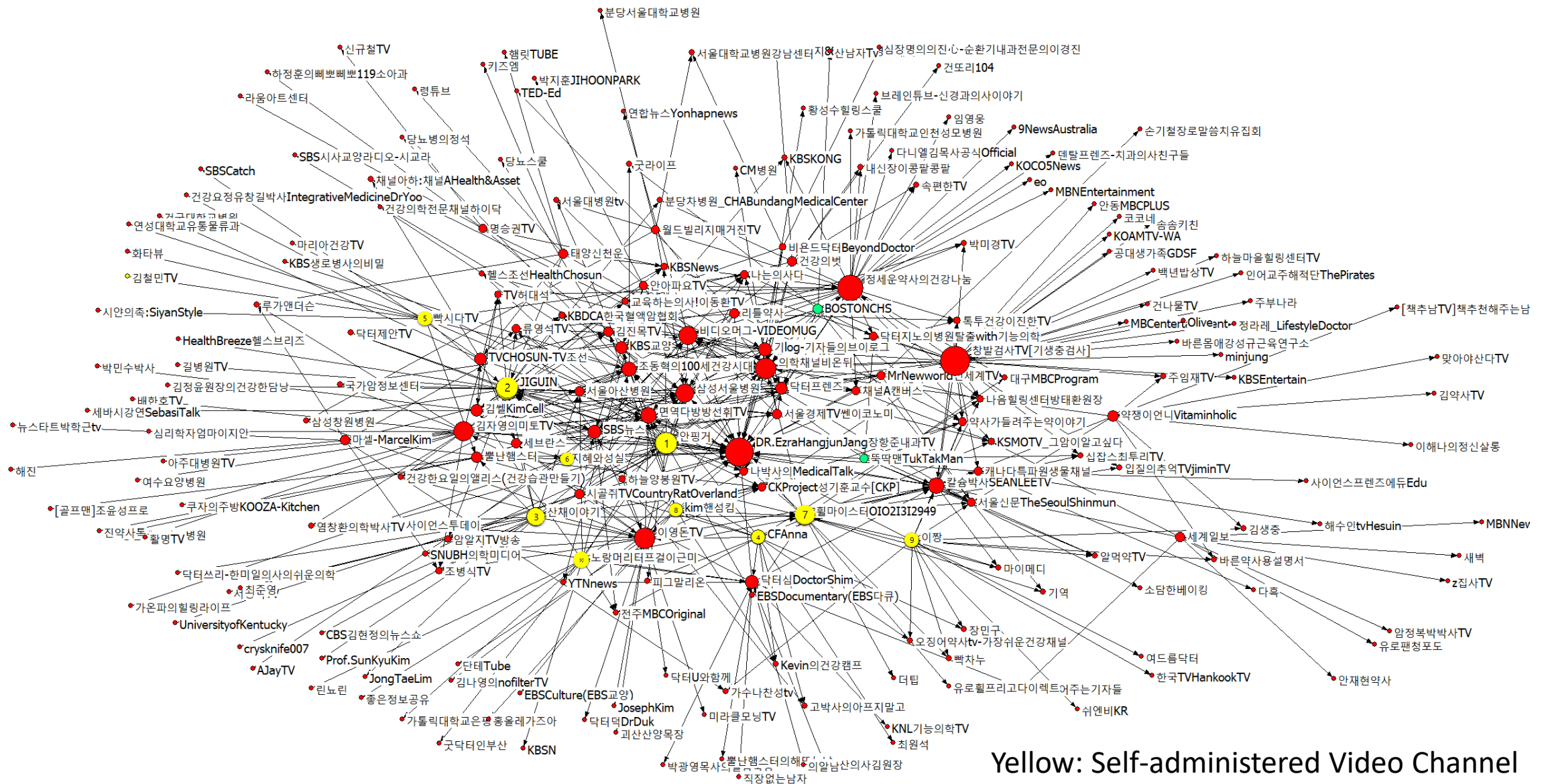

Yellow: Self-administered Video Channel

Supplement: Multimedia Appendix 1 [file jmir_v24i11e39571_app1.pdf]
